# Evaluation of oxygen-releasing mouthwash and chlorhexidine as adjuncts in gingivitis treatment: A randomized controlled trial

**DOI:** 10.6026/973206300221684

**Published:** 2026-03-31

**Authors:** Lanka Mahesh, Meenu Taneja Bhasin, Nilesh Rathi

**Affiliations:** 1 Department of Periodontology, Dr. D.Y. Patil Dental College and Hospital, Dr. D.Y. Patil Vidyapeeth, Pimpri, Pune, Maharashtra, India; 2 Department of Periodontiology, Sudha Rustagi College of Dental Sciences and Research, Faridabad, Haryana, India; 3 Department of Pediatric and Preventive Dentistry, Dr. D.Y. Patil Dental College and Hospital, Dr. D.Y. Patil Vidyapeeth, Pimpri, Pune, Maharashtra, India

**Keywords:** Bleeding on probing (BOP), chlorhexidine, gingival index, oxygen-releasing mouthwash, plaque index

## Abstract

Dental biofilm-induced gingivitis can progress to periodontitis without effective plaque control, with chlorhexidine mouthwash often used but causing side effects. Therefore, it is of interest to compare the effectiveness of oxygen-releasing and chlorhexidine mouthwashes as adjuncts to scaling and root planing (SRP). Thirty systemically healthy patients were randomized into two groups, with clinical and microbiological assessments at baseline, 14 and 30 days. Both groups showed significant improvement in clinical parameters and microbial load. The findings advance our knowledge by supporting oxygen-releasing mouthwash as a tolerable alternative to chlorhexidine with similar efficacy.

## Background:

Dental plaque induced gingivitis is a reversible inflammatory condition of the gingiva initiated by dental biofilm. It is characterized clinically by erythema, edema and bleeding on probing (BOP), without loss of periodontal attachment or radiographic bone loss [1]. The 2017 World Workshop classification emphasized the importance of accurately identifying dental biofilm-induced gingivitis and its management as a critical preventive strategy to avoid progression to periodontitis [2]. Scaling and root planing (SRP), along with meticulous oral hygiene, remain the primary approaches for managing gingivitis. Achieving and maintaining effective biofilm control, however, is often challenging due to patient-related factors and rapid plaque re-accumulation [3]. As a result, adjunctive chemical plaque control agents, such as mouthrinses, are frequently prescribed to enhance plaque control and reduce gingival inflammation during the early post-therapy period [4]. Chlorhexidine gluconate is widely regarded as the gold standard antiplaque and antigingivitis mouthwash because of its broad antimicrobial activity and substantively. Multiple trials have demonstrated its effectiveness in reducing plaque accumulation and gingival inflammation [5]. Despite its efficacy, chlorhexidine is frequently associated with adverse effects such as tooth staining, taste alteration, mucosal irritation and reduced patient acceptance. These factors limit compliance [6]. Oxygen-releasing mouthwashes have emerged as potential alternatives to chlorhexidine. These formulations increase local oxygen tension, which may inhibit the growth of obligate anaerobes and support an environment conducive to soft tissue healing [7]. Additionally, it enhances wound healing by facilitating the formation of new blood vessels, collagen synthesis and the restoration of epithelial layers. Therefore, oxygen therapy may serve as a beneficial supplement in the healing of periodontal wounds [8]. In gingivitis, although periodontal pockets are absent, microbial shifts occur within the gingival sulcus. As plaque matures, there is an increase in gram-negative anaerobes and a heightened inflammatory response [9]. Microbiological evaluation of sulcular plaque is biologically relevant even in cases of gingivitis, as it allows for objective assessment of the effects of adjunctive mouthrinses on microbial load. Several clinical studies have compared oxygenated or ozonated mouthrinses with chlorhexidine in the management of plaque-induced gingival inflammation [10]. Therefore, it is of interest to report to assess the clinical and microbiological efficacy of oxygen-releasing and chlorhexidine mouthwashes as adjuncts to SRP in patients with dental biofilm-induced gingivitis, while also evaluating patient-reported outcomes regarding taste perception, oral comfort, tolerability and compliance.

## Methodology:

The present investigation was conducted as a prospective, randomized, interventional in vivo clinical and microbiological trial to evaluate and compare the efficacy of an oxygen-releasing mouthwash and a chlorhexidine mouthwash as adjuncts to SRP in patients with dental biofilm-induced gingivitis. Participants were selected from the institution's outpatient department based on predefined inclusion criteria. Ethical approval was granted by the Institutional Ethics Committee, SRCDSR, Faridabad and the study protocol adhered to the principles of the Declaration of Helsinki. Written informed consent was obtained from all participants prior to enrolment. Systemically healthy patients diagnosed with dental biofilm-induced gingivitis, according to the 2017 World Workshop classification, possess at least 20 natural teeth and demonstrate willingness to comply with the study protocol were recruited from the outpatient department of Periodontology. Diagnosis was established through clinical examination, which identified gingival inflammation and BOP without evidence of clinical attachment loss or radiographic bone loss. Exclusion criteria included a history of periodontal therapy within the previous six months, recent use of antibiotics or anti-inflammatory drugs, tobacco use, pregnancy or lactation and systemic conditions known to affect periodontal health. The enrolled subjects were randomly allocated into two groups using the sequentially numbered opaque sealed envelope method. The test group received SRP followed by the use of an oxygen-releasing blue®M mouthwash, while the control group received SRP with CHX mouthwash. All participants received full-mouth scaling with ultrasonic scalers and hand instruments. Standardized oral hygiene instructions were provided and reinforced for each patient. Participants were instructed to rinse with 10 mL of the assigned mouthwash twice daily for one minute, beginning 30 minutes after tooth brushing and to abstain from eating or drinking for at least 30 minutes following rinsing. The mouthwash regimen was maintained for 14 days. Clinical parameters were recorded by a single calibrated examiner at baseline, 14 days and 30 days. The clinical indices assessed included Plaque Index (PI) (Silness and Löe), Gingival Index (GI) (Löe and Silness) and BOP. Subgingival plaque samples were collected from the gingival sulcus of the most inflamed site using sterile curettes at baseline and at 30 days. Although gingivitis does not result in periodontal pockets, sampling from the gingival sulcus remains microbiologically relevant because gingival inflammation is linked to increased microbial load and compositional changes within the sulcular biofilm. Samples were transferred to appropriate transport media and cultured under suitable laboratory conditions. Microbial load was quantified by counting colony-forming units (CFU). The collected data were analyzed using appropriate statistical software. Descriptive statistics were calculated for all clinical and microbiological parameters and expressed as mean ± standard deviation. Intragroup comparisons were performed to assess changes in PI, GI and BOP scores over time (baseline, 14 days and 30 days), as well as changes in sulcular microbial load (CFU) between baseline and 30 days, using the paired t-test. Intergroup comparisons between the oxygen-releasing mouthwash group and the chlorhexidine mouthwash group at corresponding time intervals were carried out using the independent t-test. A p-value of <0.05 was considered statistically significant.

## Results:

This study aimed to compare the clinical and microbiological efficacy of oxygen-releasing mouthwash (Blue®M) and chlorhexidine (CHX) as adjuncts to SRP in patients with dental biofilm-induced gingivitis. The demographic characteristics of the participants showed no significant differences between the groups in terms of age (32.5 ± 6.2 for CHX, 33.1 ± 5.8 for Blue®M) and gender distribution, with both groups being comparable (p < 0.05) (Table 1 - see PDF). Regarding clinical outcomes, both mouthwashes led to a significant reduction in PI scores over the course of the study. At baseline, the CHX group had a PI score of 2.5 ± 0.3 and the Blue®M group had 2.4 ± 0.2. By Day 30, the scores had reduced to 1.4 ± 0.3 for CHX and 1.3 ± 0.2 for Blue®M. The reductions were statistically significant in both groups at each time point (p < 0.05), (Table 2, Figure 1 - see PDF). A similar trend was observed for GI scores, which decreased significantly from baseline to Day 30. For instance, the CHX group had a baseline GI of 2.3 ± 0.2, which reduced to 1.2 ± 0.2 by Day 30. The Blue®M group also showed a comparable reduction from 2.2 ± 0.3 to 1.1 ± 0.2 (p < 0.05), as illustrated in (Table 3, Figure 2 - see PDF). BOP scores, a sensitive marker of gingival inflammation, also demonstrated significant improvement, with reductions noted at Day 14 and Day 30 in both groups shown in (Table 4, Figure 3 - see PDF). Microbiological analysis revealed that both mouthwashes were effective in reducing the microbial load in the gingival sulcus. At baseline, the CHX group had a microbial count of 6.8 x 10^6^ ± 1.2 x 10^6^ CFU, while the Blue®M group had 6.5 x 10^6^ ± 1.1 x 10^6^ CFU. By Day 30, the microbial counts in both groups had significantly decreased to 2.5 x 10^3^ ± 1.0 x 10^3^ CFU for CHX and 2.2 x 10^3^ ± 0.9 x 10^3^ CFU for Blue®M (Table 5 - see PDF), with both reductions being statistically significant (p < 0.05). In terms of patient-reported outcomes, the oxygen-releasing mouthwash group showed better overall tolerability and comfort. The Chlorhexidine group experienced adverse effects such as taste alteration in 12 patients and tooth staining in 10 patients, which is consistent with previous studies. In contrast, no adverse effects were noted in 14 patients in the Blue®M group. Additionally, 14 patients in the Blue®M group reported improved oral comfort, whereas 8 patients in the CHX group reported reduced comfort. Tolerability was rated as poor in 7 patients using CHX but was rated as good in 14 patients using Blue®M (Table 6 - see PDF). Overall, the study demonstrates that both mouthwashes resulted in significant improvements in clinical and microbiological parameters in patients with dental biofilm-induced gingivitis. However, the oxygen-releasing mouthwash was better tolerated, with fewer adverse effects, making it a promising alternative to Chlorhexidine in the management of gingivitis.

## Discussion:

Sindhusha and Rajasekar (2023) [11] found that using an oxygen-enriched mouthwash (Blue®M) as a pre-procedural rinse significantly reduced bacterial load in aerosols produced during ultrasonic scaling compared with chlorhexidine, indicating superior microbial reduction in aerosolized bacterial contamination under clinical conditions. Kumar *et al.* (2024) [12] reported that in patients with plaque induced gingivitis, Blue®M mouthwash showed comparable reductions in GI and BOP to chlorhexidine and even greater PI reduction at 1 month, aligning with our findings on clinical improvements. Teixeira *et al.* (2025) [13] conducted a randomized trial in intubated intensive care unit (ICU) patients showing that Blue®M and chlorhexidine mouthwashes were similarly effective in reducing oral microbial populations, supporting our conclusion that the oxygen releasing mouthwash has equivalent antimicrobial effects. Niveda & Kaarthikeyan (2020) [14] compared an oxygen releasing gel (similar therapeutic mechanism to oxygen releasing rinse) with chlorhexidine gel in the treatment of periodontal pockets and found a greater reduction in probing depth with oxygen releasing therapy, suggesting beneficial clinical effects of topical oxygen agents as adjuncts. In this study, both oxygen-releasing and chlorhexidine mouthwashes demonstrated significant improvements in clinical and microbiological parameters in patients with dental biofilm- induced gingivitis. While both treatments showed comparable efficacy in plaque reduction, gingival inflammation and microbial load, the oxygen-releasing mouthwash was better tolerated with fewer adverse effects. These findings suggest that oxygen-releasing mouthwash is a viable alternative to chlorhexidine for managing gingivitis. Future studies with longer follow-up and more specific microbial analyses could further elucidate the long-term benefits of oxygen-releasing mouthwashes.

## Conclusion:

In conclusion, both oxygen-releasing and chlorhexidine mouthwashes significantly improved clinical and microbiological outcomes in patients with dental biofilm-induced gingivitis. The oxygen-releasing mouthwash demonstrated similar efficacy to chlorhexidine but with better tolerability. Therefore, oxygen-releasing mouthwash may be a viable alternative to chlorhexidine for managing gingivitis.
